# The Kinematic and Kinetic Development of Sprinting and Countermovement Jump Performance in Boys

**DOI:** 10.3389/fbioe.2020.547075

**Published:** 2020-11-05

**Authors:** Maximilian M. Wdowski, Mark Noon, Peter D. Mundy, Marianne J. R. Gittoes, Michael J. Duncan

**Affiliations:** ^1^Faculty of Health and Life Sciences, Coventry University, Coventry, United Kingdom; ^2^Centre for Sport, Exercise and Life Sciences, Coventry University, Coventry, United Kingdom; ^3^Cardiff School of Sport and Health Sciences, Cardiff Metropolitan University, Cardiff, United Kingdom

**Keywords:** children, acceleration, biomechanics, jumping, functional movements, fundamental movement skill, motor competence

## Abstract

**Background:**

The aim of the study was to examine the kinematics and kinetics of sprint running and countermovement jump performance between the ages of 8–9, and 11–12 years old boys in order to understand the developmental plateau in performance.

**Methods:**

18 physically active boys (Age: 10.1 ± 1.6), in an under 9 years old (U9) and an under 12 years old (U12) group performed 15 m sprints and countermovement jumps. A 3D motion analysis system (200 Hz), synchronized with four force platforms (1,000 Hz), was used to collect kinematic and kinetic data during the first stance phase of the sprint run and the countermovement jump.

**Results:**

The U12 group had a significantly greater height (U9: 1.364 ± 0.064 m; U12: 1.548 ± 0.046 mm), larger mass (U9: 30.9 ± 3.5 kg; U12: 43.9 ± 5.0 kg), superior sprint performance over 0–5 m (U9: 1.31 ± 0.007 s; U12: 1.23 ± 0.009 s) and 0–15 m (U9: 3.20 ± 0.17 s; U12: 3.01 ± 0.20 s), and increased jump height (U9: 0.17 ± 0.06 m; U12: 0.24 ± 0.10 m) than the under nine group. During the first stance phase of the sprint the U12 group had a significantly greater vertical (U9: 0.22 ± 0.02 BW/s; U12: 0.25 ± 0.03 BW.s) and horizontal impulse (U9: 0.07 ± 0.02 BW/s; U12: 0.09 ± 0.03 BW.s) than the U9 group. When performing a countermovement jump the U12 group had a significantly greater mean average eccentric force (U9: 407.3 ± 55.0 N; U12: 542.2 ± 65.1 N) and mean average concentric force (U9: 495.8 ± 41.3 N; U12: 684.0 ± 62.1 N). Joint kinematics for the countermovement jump were significantly different between age groups for the ankle range of motion (U9: 80.6 ± 17.4°; U12: 64.1 ± 9°) and knee minimum joint angle (U9: −5.7 ± 3.9°; U12: 0.0 ± 4.4°). Conclusion: The study demonstrates for the first time that the development of physically active boys between the ages of 8–9 to 11–12 years increased the ground reaction forces and impulses during sprint running and countermovement jumps, but that sprint running technique had not developed during this period. Furthermore, countermovement jump technique was still emerging at the age of 8–9 years old. Practitioners need to implement on-going fine-grained sprint running and CMJ technique sessions to ensure that the increased force producing capabilities that come with age are appropriately utilized.

## Introduction

Successful performance of rapid movements such as sprints and jumps are essential in many sports, particularly team sports (Little and Williams, 2005; Gabbett et al., 2008; Salaj and Markovic, 2011). Sprinting and jumping ability are fundamental locomotive skills that form key aspects of athletic motor skill (Lloyd et al., 2015) whilst also being recognized as some of the key fundamental movement skills needed for children to lead physically active lives (Duncan et al., 2020). Furthermore, they are commonly used within talent identification, selection and development to differentiate between potential elite and non-elite youth athletes (Gissis et al., 2006; Till et al., 2017; Sarmento et al., 2018).

Sprint performance has been generally measured in children using stopwatches and timing gates (Le Gall et al., 2002**;** Kotzamanidis, 2006), where such measures have been proven reliable with intra- and inter-day intraclass correlation coefficients (ICC) ranging from 0.88 to 0.98 for 10–40 m sprints (Christou et al., 2006; Kotzamanidis, 2006). Maximal effort sprinting and CMJ performance have been shown to increase with childhood development (Viru et al., 1999; Meyers et al., 2016). There has been an increased interest in the use of force plates to collect countermovement jump (CMJ) variables, potentially because of their increase in affordability and accessibility (Chavda et al., 2017). With such equipment, jump height has been shown to be calculated reliably between 9 and 16 years of age (Meylan et al., 2012).

The Football Association, 2019 Handbook (The FA Handbook 2019/2020) defines those participating at ages under 7 years old to under 10 years old as playing “Mini Soccer” where the matches develop from 5-aside to 7-aside. There is then a progression to “Youth Football” for those participating at ages under 11 years old to under 18 years old, where matches develop from 7-aside to full 11-aside. It has been observed that sprint and CMJ performance in boys rapidly develops from 5 years to 14 years of age (Viru et al., 1999), but that there is a plateau in both sprint and CMJ performance from approximately nine years to 12 years of age (Yague and De La Fuente, 1998; Philippaerts et al., 2006; Focke et al., 2013; Meyers et al., 2015, 2016; Nagahara et al., 2018). This slower development of performance has been suggested to occur around 1.5–2.5 years before peak height velocity (PHV) in boys, which usually happens around the age of 13 or 14 years old (Yague and De La Fuente, 1998; Philippaerts et al., 2006; Meyers et al., 2015, 2016). The main arguments for this plateau in performance are often associated with the central nervous system development, supported by the rapid growth of the central nervous system during the first 7 years of life (Malina et al., 2004), and the opinion that coordination patterns of locomotor skills reach adult levels by the same age (Whithall, 2003), the development of neural control in jumping (Oliver and Smith, 2010) and the potential phenomenon of adolescent awkwardness (Philippaerts et al., 2006). Therefore, understanding any differences in kinematic and kinetic variables of sprint running and CMJ performance across children of different developmental stages is key in the comprehension of the plateau in performance.

Technique describes the relative position and orientation of body segments as they change during the performance of a sport task to perform that task effectively (Lees, 2002). Previous literature has used joint angular motion to provide novel insights regarding specific kinematic features of technique (Miyamoto et al., 2018) and kinetics to understand elements of technique that may influence the ground reaction force vector (GRF) (Bezodis et al., 2016). At present there are only a few studies that have investigated sprint running or CMJ technique during the plateau of performance in boys. One study by Nagahara et al. (2018) investigated the kinetics of the stance phase and found that an unchanged propulsive force characterized the developmental plateau in performance and lower SF during all phases of a 50.5 m maximal sprint run. However, the study did not investigate joint kinematics during the sprint performance so could not provide a deeper understanding into the technical effects of slower development in sprinting ability. A further study by Focke et al. (2013) on the kinetics of the CMJ in children and adolescents highlighted a period of limited development in jump height when normalized to body height between 8 and 12 years of age. They suggested that the increase in absolute jump height during this period was due to an increase in leg lengths, leg muscle volumes and muscle forces. Similar to Focke et al. (2013), Nagahara et al. (2018) did not investigate the movement patterns of joints during the performance that might provide extended insight into technical effects during the period of slower development in CMJ performance. No study to date has examined kinetics and kinematics of sprint running or CMJ performance in children. Combining both kinematic and kinetic assessment is a key step forward in understanding the development of movement in children. Without such information strength and conditioning coaches, physical educationalists and coaches have no evidence base upon which to build their interventions.

Therefore, the aim of the study was to examine the kinematics and kinetics of sprint running and CMJ performance between the ages of 8–9, and 11–12 years old boys. This study will provide critical information regarding the development of sprinting and CMJ performance during this important developmental stage in order to inform the potential development of youth training and talent identification programs in sport. It was hypothesized that the older age group would ascertain a higher level of performance as a consequence of applying more force during the sprinting and CMJ performances than the younger age group. It was further hypothesized that sprinting and CMJ kinematics would be different between groups, as the older group would have superior technique efficacy.

## Materials and Methods

### Participants

Eighteen physically active boys were recruited for the study (Age: 10.1 ± 1.6). In order to ensure training volume, type and competitive level homogeneity, the participants were recruited through the same soccer club where they engaged in twice-weekly soccer training and further once weekly match play. Training sessions were skill based and lasted 1 h, with the match play performed in accordance with their age group regulations (The FA Handbook 2019/2020). The participants were separated into two groups of ten 8–9 years olds (U9) (Age: 8.7 ± 0.5) and eight 11–12 years olds (U12) (Age: 11.8 ± 0.5). Signed informed consent was obtained from legal guardians, and verbal assent was obtained from children using the procedures approved by the ethics governance procedure at Coventry University. Prior to data collection, the participant’s parent/guardian verified that they had no musculoskeletal impediment that would impede movement.

### Experimental Protocol and Data Collection

Participants’ mass (kilograms), height and right and left leg lengths were measured. Leg length was measured with the participant lying supine from the bony landmark of the anterior superior iliac spine to the lateral malleolus of the ankle. A handheld Vernier Caliper (Draper Tools Ltd., United Kingdom) was used to measure right and left knee and ankle widths at the widest point across the medial and lateral condyles and malleoli, respectively. Leg length to height ratio (LLHR) was calculated as the ratio of leg length to body height: (leg-length/height) × 100 (Liu et al., 2014).

Sixteen 14 mm reflective markers (Vicon Lower-Limb Plug-in-Gait Marker Set, 2019) were placed over the following anatomical locations bilaterally: second metatarsal head, calcaneus, lateral malleolus, lateral shank, lateral epicondyle, lateral thigh, anterior superior iliac spine, and posterior superior iliac spine. The positions of the reflective markers during the sprint acceleration runs and CMJ’s were recorded at 200 Hz using a 12-camera Vicon Vantage 3-D motion capture system (Vicon Nexus 2.0, Oxford, United Kingdom).

After completing a static trial (as instructed in the Vicon Plug-In-Gait user guide, accessed 01/06/2019) participants then performed three maximal 15 m sprint acceleration runs from a standing start and were free to choose their front leg in this stance (Lockie et al., 2013). Photoelectric timing gates (SmartSpeed, FusionSport, United Kingdom) were used to collect interval sprint time data over 0–5 and 0–15 m. The participants were positioned 0.3 m back from the first photoelectric timing gate at the start line (Oliver and Meyers, 2009). The start line was positioned 0.5 m posteriorly back from the start of a 0.90 × 0.60 force platform (AMTI OR6 Series Force Plate, United States, 1,000 Hz), mounted underneath an athletic track surface, so that the first foot strike would contact near the center (Wdowski and Gittoes, 2020). The fastest sprint running trial according to the 0–15 m sprint times where contact was made with the center of the force platform was used for further analysis.

Participants then performed three countermovement vertical jump trials. The countermovement jump consisted of two phases. In the eccentric phase, the center of mass is lowered by flexing the hips, knees, and ankles (dorsiflexion). The propulsive phase begins when the descent of the center of mass stops, generally at maximum knee flexion. From the crouched position, extension of the hips, knees, and plantar flexion of the ankles propels the body upward until the feet leave the ground (takeoff) (Cowley et al., 2020). The participants stood with each foot on a separate 0.90 × 0.60 force platform (AMTI OR6 Series Force Plate, United States, 1,000 Hz) mounted underneath an athletic track surface. Each trial began with the participant standing still. An investigator in sight of the participant gave the prompt to jump and the participant performed a maximum-effort CMJ, with their hands placed on their iliac crest. No other verbal instructions were provided, and participants were not instructed in jumping technique. A successful trial was classified as the participant standing stationary with both feet on the floor, jumping straight up, and landing on both feet without taking any steps. The trial with the largest jump height was used for further analysis.

### Data Processing

All marker trajectory data were filtered using a fourth-order, zero-lag, Butterworth filter with a cut-off frequency of 10 Hz. Sagittal plane kinematics (relative joint angle and joint angular velocity) of the right ankle, knee, hip, and pelvis joint were calculated using the Vicon plug-in-gait model. Force data were filtered using a fourth-order, zero-lag, Butterworth filter with a cut-off frequency of 200 Hz. For sprint running the instants of touchdown and take-off from the force plate were defined when the vertical GRF first rose above 10 N (touchdown) and declined below 10 N (take-off). This period from touchdown to take-off was then defined as the contact time. Vertical (Z), anteroposterior (Y) and medial-lateral (X) GRF data were then exported for the duration of the contact time for each participant and normalized to Newton’s\body weight (BW). Peak and mean average GRFs during stance were identified and GRF impulses were determined using the trapezium rule of integration. Orientation of ground reaction force vectors for the YZ comparison were calculated using the procedures outlined by Morin et al. (2011). Kinematic variables for the sagittal plane during the contact time for the swing and stance legs were identified as the ankle, knee and hip joint angle and at touchdown and toe-off, as well as, the range of motion during the contact time, and peak angular velocity of extension and flexion at the knee, hip, and ankle (plantar flexion and dorsiflexion).

The GRF data from the two separate force platforms for the CMJ were added together and the jump height and key CMJ kinetic variables were calculated using the method of Chavda et al. (2017). Kinematic variables were calculated from the moment that the eccentric phase was initiated (the moment the pelvis markers began to movement vertically downwards) to take-off (when force declined below 10 N). The kinematic variables identified were the ankle, knee and hip joint angular range of motion, as well as, peak angular velocity of extension during the propulsive phase and flexion during the countermovement phase at the knee, hip, and ankle (Plantar flexion and dorsiflexion).

### Data Analysis

For each variable, normality was established with the Shapiro–Wilk test, and because of the assumption of normality being violated, between age group means were compared using the Mann–Whitney nonparametric test. Significance was set at *P* < 0.05. Effect sizes were calculated using Cohen’s d (Cohen, 1988). All statistical analyses were carried out using SPSS Statistics for Windows (version 24.0; IBM SPSS, United States). A step-wise multiple linear regression was conducted to observe if the key performance outcome, kinematic and kinetic variables of sprint running and CMJ predicted the age of the participant.

## Results

The U12 group had significantly different anthropometric variables to the U9 group (Table 1). In general, the U12 group had increased by approximately 8% across all statistically significant anthropometric measures. For example, the height, body mass, leg length, knee width, and ankle width increased by 7.3–8.8% (*P* = 0.000–0.003; *d* = −1.35 to −1.88).

**TABLE 1:** Anthropometric variables for the 8–9 years old boys and the 11–12 years old boys.

	**8–9 years**	**11–12 years**	***P***	**Cohen’s d**
Height (m)	1.364 ± 0.064	1.548 ± 0.046	0.000*	−1.88
Mass (kg)	30.9 ± 3.9	43.9 ± 5.0	0.000*	−1.67
Leg length (cm)	70.9 ± 4.4	80.6 ± 5.5	0.001*	−1.41
Ankle width (cm)	6.1 ± 0.5	6.9 ± 0.3	0.002*	−1.35
Knee width (cm)	8.3 ± 0.5	9.4 ± 0.4	0.000*	−1.59
LLHR (%)	51.8 ± 1.8	52.1 ± 2.7	0.897	−0.15

*Significant difference between age groups.

The U12 group displayed a 6.1 % shorter sprint time over the 0–5 m (*P* = 0.043; *d* = 0.92) and 5.9% over the 0–15 m (*P* = 0.034; *d* = 0.91) when compared to the U9 group (Table 2). During the first stance phase of the sprint the U12 group had a significantly greater vertical (U9: 0.22 ± 0.02 BW.s; U12: 0.25 ± 0.03 BW.s; *P* = 0.027; *d* = −1.09) and horizontal impulse (U9: 0.07 ± 0.02 BW.s; U12: 0.09 ± 0.03 BW.s; *P* = 0.043; *d* = −0.82) than the U9 group. No differences in sprint running kinematics were observed between age groups. However, large effects were observed for a reduction in the stance knee flexion (U9: 602.2 ± 304.9°/s; U12: 324.7 ± 139.0°/s; *P* = 0.083; *d* = 1.00) and ankle dorsiflexion (U9: 1144.2 ± 776.0°/s; U12: 600.5 ± 249.2°/s; *P* = 0.068; *d* = 0.84) angular velocity (°/s) for the U12 group when compared to the U9 group.

**TABLE 2:** Sprint running performance, kinematic and kinetic variables for the 8–9 years old boys and the 11–12 years old boys.

	**8–9 years**	**11–12 years**	***P***	**Cohen’s d**
0–5 m time (s)	1.31 ± 0.07	1.23 ± 0.09	0.043*	0.92
0–15 m time (s)	3.20 ± 0.17	3.01 ± 0.20	0.034*	0.91
Peak GRF Y (BW)	0.62 ± 0.14	0.74 ± 0.11	0.068	−0.87
Average GRF Y (BW)	0.35 ± 0.09	0.40 ± 0.08	0.083	−0.64
Impulse Y (BW.s)	0.07 ± 0.02	0.09 ± 0.03	0.043*	−0.82
Peak GRF Z (BW)	1.82 ± 0.25	1.93 ± 0.24	0.146	−0.47
Average GRF Z (BW)	1.08 ± 0.13	1.15 ± 0.12	0.237	−0.60
Impulse Z (BW.s)	0.22 ± 0.02	0.25 ± 0.03	0.027*	−1.09
Average YZ orientation angle (°)	17.4 ± 3.3	18.6 ± 5.9	0.859	−0.25
Contact Time (s)	0.21 ± 0.02	0.22 ± 0.03	0.460	−0.54
Stance ankle at TD (°)	16.8 ± 3.8	15.4 ± 5.4	0.696	0.32
Stance knee at TD (°)	53.2 ± 8.0	58.0 ± 18.6	0.274	−0.35
Stance hip at TD (°)	61.2 ± 10.3	59.3 ± 8.8	0.515	0.20
Pelvis at TD (°)	35.2 ± 5.8	33.1 ± 5.4	0.573	0.37
Stance ankle at TO (°)	−20.0 ± 11.8	-17.8 ± 10.2	0.633	−0.21
Stance knee at TO (°)	15.0 ± 9.4	16.7 ± 6.5	0.173	−0.22
Stance hip at TO (°)	1.0 ± 7.0	0.7 ± 5.5	0.897	0.05
Pelvis at TO (°)	35.0 ± 5.1	32.5 ± 5.6	0.360	0.47
Stance ankle ROM (°)	55.1 ± 14.6	50.4 ± 12.1	0.633	0.35
Stance ankle plantarflexion angular velocity (°.s)	−1418.2 ± 877.6	−956.5 ± 270.1	0.203	−0.66
Stance ankle dorsiflexion angular velocity (°.s)	1144.2 ± 776.0	600.5 ± 249.2	0.068	0.84
Stance knee ROM (°)	42.6 ± 8.3	44.6 ± 13.4	0.573	−0.19
Stance knee angular extension velocity (°.s)	−649.4 ± 360.9	−556.2 ± 165.6	0.897	−0.32
Stance knee angular flexion velocity (°.s)	602.2 ± 304.9	324.7 ± 139.0	0.083	1.00
Stance hip ROM (°)	60.8 ± 7.4	58.6 ± 8.5	0.829	0.28
Stance hip angular extension velocity (°.s)	−499.5 ± 68.2	−473.7 ± 100.1	0.573	−0.31
Stance hip angular flexion velocity (°.s)	35.9 ± 150.3	8.0 ± 76.2	0.829	0.23
Pelvis ROM (°)	5.3 ± 2.0	7.3 ± 3.3	0.237	−0.72
Swing ankle ROM (°)	36.1 ± 5.3	34.2 ± 5.7	0.762	0.35
Swing ankle plantarflexion angular velocity (°.s)	−203.4 ± 245.4	−156.9 ± 140.7	0.762	−0.23
Swing ankle dorsiflexion angular velocity (°.s)	363.4 ± 54.2	383.0 ± 151.9	0.573	−0.19
Swing knee ROM (°)	70.9 ± 10.7	74.7 ± 8.9	0.515	−0.38
Swing knee angular extension velocity (°.s)	−761.8 ± 251.5	−638.0 ± 207.0	0.237	−0.53
Swing knee angular flexion velocity (°.s)	880.8 ± 266.5	766.2 ± 198.6	0.408	0.48
Swing hip ROM (°)	69.4 ± 8.9	70.6 ± 9.5	0.897	−0.14
Swing hip extension angular velocity (°.s)	−218.6 ± 163.5	−211.4 ± 109.8	0.573	−0.05
Swing hip flexion angular velocity (°.s)	681.4 ± 80.8	617.3 ± 70.4	0.122	0.79

*Significant difference between age groups.

The U12 group were observed (Table 3) to jump 41.2% higher than the U9 group when performing a CMJ (*P* = 0.043; *d* = −0.84). During the CMJ, the U12 group had a significantly greater mean average eccentric force (U9: 1.26 ± 0.17 BW; U12: 1.68 ± 0.20 BW; *P* = 0.001; *d* = −1.50) and mean average concentric force (U9: 1.53 ± 0.13 BW; U12: 2.11 ± 0.19 BW; *P* = 0.000; *d* = −1.74). Joint kinematics for the CMJ were observed to be significantly different between age groups for the ankle maximum plantarflexion (U9: −43.2 ± 10.8°; U12: −29.3 ± 10.8°; *P* = 0.012; *d* = −1.10), ankle range of motion (U9: 80.6 ± 17.4°; U12: 64.1 ± 9°; *P* = 0.034; *d* = 1.02) and knee maximum joint extension (U9: −5.7 ± 3.9°; U12: 0.0 ± 4.4°; *P* = 0.021; *d* = −1.15).

**TABLE 3:** CMJ performance, kinematic and kinetic variables for the 8–9 years old boys and the 11–12 years old boys.

	**8–9 years**	**11–12 years**	***P***	**Cohen’s d**
Jump height (m)	0.17 ± 0.06	0.24 ± 0.10	0.043*	−0.84
Max eccentric force (BW)	1.61 ± 0.28	2.10 ± 0.32	0.002*	−1.29
Average eccentric force (BW)	1.26 ± 0.17	1.68 ± 0.20	0.001*	−1.50
Average eccentric velocity (m/s)	−0.44 ± 0.16	−0.43 ± 0.16	0.633	−0.10
Maximum eccentric power (W/BW)	−0.71 ± 0.29	−0.91 ± 0.43	0.460	0.57
Average eccentric power (W/BW)	−0.52 ± 0.22	−0.67 ± 0.34	0.408	−0.60
Eccentric time (s)	0.22 ± 0.06	0.24 ± 0.09	0.762	−0.23
Max concentric force (BW)	2.00 ± 0.21	2.74 ± 0.38	0.000*	−1.56
Average concentric force (BW)	1.53 ± 0.13	2.11 ± 0.19	0.000*	−1.74
Maximum concentric velocity (m/s)	2.06 ± 0.32	2.41 ± 0.41	0.043*	−0.90
Average concentric velocity (m/s)	1.20 ± 0.14	1.31 ± 0.20	0.173	−0.65
Max concentric power (W/BW)	3.27 ± 0.48	5.48 ± 1.06	0.000*	−1.61
Average concentric power BW)	1.65 ± 0.20	2.57 ± 0.44	0.000*	−1.62
Concentric time (s)	0.29 ± 0.09	0.35 ± 0.10	0.055	−0.61
Propulsion time (s)	0.25 ± 0.09	0.31 ± 0.09	0.034*	−0.67
Ankle maximum plantarflexion (°)	−43.2 ± 10.8	−29.3 ± 10.8	0.012*	−1.10
Ankle maximum dorsiflexion (°)	37.4 ± 8.9	34.8 ± 7.6	0.460	0.32
Ankle ROM (°)	80.6 ± 17.4	64.1 ± 9.0	0.034*	1.02
Ankle plantarflexion angular velocity (°.s)	−1301.7 ± 362.6	−889.8 ± 254.8	0.012*	−1.10
Ankle dorsiflexion angular velocity (°.s)	150.3 ± 105.3	134.0 ± 50.0	1.000	0.19
Knee maximum extension (°)	−5.7 ± 3.9	0.0 ± 4.4	0.021*	−1.15
Knee maximum flexion (°)	94.1 ± 20.1	91.7 ± 19.8	0.829	0.13
Knee ROM (°)	99.8 ± 19.2	91.6 ± 17.6	0.360	0.44
Knee extension angular velocity (°.s)	−1024.7 ± 154.2	−880.2 ± 143.6	0.146	−0.89
Knee flexion angular velocity (°.s)	267.6 ± 115.5	231.8 ± 73.5	0.360	0.37
Hip maximum extension(°)	7.7 ± 3.7	6.0 ± 5.0	0.237	0.42
Hip maximum flexion (°)	87.9 ± 8.9	88.1 ± 12.6	0.829	−0.02
Hip ROM (°)	80.2 ± 9.5	82.1 ± 13.0	0.573	−0.18
Hip extension angular velocity (°.s)	−599.8 ± 94.2	−575.3 ± 94.1	0.829	0.17
Hip flexion angular velocity (°.s)	252.6 ± 92.2	215.0 ± 87.9	0.360	0.42

*Significant difference between age groups.

A stepwise multiple linear regression was calculated to predict the participants’ age based on the significant performance outcome, anthropometric, kinetic and kinematic variables of sprint running and CMJ performance. A significant regression equation was found [*F*_(2, 15)_ = 127.081, *P* = 0.0000] with an *R*^2^ of 0.944 and an adjusted *R*^2^ of 0.937. Participants predicted age is equal to 9.013 − 5.036 (0–5 m time) + 4.180 (Average concentric force BW), where 0–5 m time is measured in time, and average concentric force is measured in Newton’s\body weight (BW).

## Discussion

The aim of the study was to examine the kinematics and kinetics of sprint running and CMJ performance between the ages of 8–9, and 11–12 years old boys. This is the first study to concurrently examine the kinetics and kinematics of acceleration sprint running and the CMJ during the important developmental ages of 8 and 12 in children. The findings are therefore unique and have practical applications for sport and exercise scientists, physical educators and strength and conditioning coaches who work with children to enhance movement performance, selection and talent identification across a range of sports. We found large differences in acceleration sprint performance over 0–5 and 0–15 m, and in CMJ height performance between the age groups supporting our hypothesis. Furthermore, the superior performance was underpinned by an increase in the application of force and impulse during CMJ and sprint accelerations, respectively. Such findings support a growing body of literature, based on assessment on kinetics only, investigating the force production during sprint running and CMJ in children and adolescents (Focke et al., 2013; Nagahara et al., 2018). The added novelty of joint kinematics in the current study elucidated that differences in sprint running performance were not because of adapted sprint running technique, and that superior CMJ performance was partly attributed to developed movement technique during the CMJ. The findings might highlight to coaches and practitioners the need for on-going fine-grained sprint running and CMJ technique sessions to ensure that the increased force producing capabilities that come with age are appropriately utilized.

Running velocity is an important component in many sports (Rumpf et al., 2019). The older group of boys were 9% faster than the younger group over both the 0–5 and 0–15 m sprint times. Previous research by Nagahara et al. (2018) found similar differences between age groups of under 8.8 years, between 8.8 and 12.1 years and over 12.1 years for the initial four steps and middle acceleration period. The peak horizontal GRF in the U9 (0.62 ± 0.14 BW) and U12 (0.74 ± 0.11 BW) was found to be approximately 50% lower than elite adult sprinters (1.2 BW) (Bezodis et al., 2014). It was observed that superior horizontal and vertical impulse during the first stance phase of an acceleration sprint run in the U12 group when compared to the U9 group. The larger horizontal impulse provided to the ground during the first stance phase by the U12 group was a potential key contributor to superior acceleration sprint performance. Mechanically, a greater change in running velocity is achieved by greater mean net anterior–posterior force and a larger relative anteroposterior impulse (Hunter et al., 2005; Morin et al., 2015; Rabita et al., 2015). The older group also applied an increased vertical impulse to the ground during the first stance phase. The increased vertical impulse has been suggested, along with having a higher height, to contribute to an increased step length (Nagahara et al., 2018). Deterministic models of sprint performance have previously suggested that an increase in the step length would result in a greater sprint velocity (Hunter et al., 2004). Therefore, during the developmental stage of 9–12 years old, boys could have increased their horizontal and vertical GRF impulse when executing an acceleration sprint to improve sprint performance. Future research into fine-grained step-by-step analysis of a sprint run during this developmental stage would advance this understanding.

The kinematic analysis of the first stance phase of an acceleration sprint performance suggested no significant technique changes to the kinematics of the movement between the U9 and U12 groups. The main arguments for a plateau in performance between the ages of 9–12 years old in boys are often associated with the central nervous system development (Malina et al., 2004), the development of neural control in jumping (Oliver and Smith, 2010) and the potential phenomenon of adolescent awkwardness (Philippaerts et al., 2006). With regard to sprinting performance, running is one of the most fundamental motor skills, which typically emerges around two years after the birth (Gallahue, 1982). Upon reaching the age of 6–7 years, the running movement of children exhibits many of the same spatio-temporal characteristics as adults (Miyamaru, 2001). Therefore, without targeting sprint technique coaching, our findings potentially introduce the concept that the development of sprint running technique may have already plateaued in physically active boys by the age of 8–9 years old, whereas in other populations such as sedentary children it may not be the case. Practitioners may benefit from introducing targeted sprint technique training sessions to improve sprint technical efficacy in physically active boys of this age.

Although no significant differences were found between age groups, large effects between groups were observed in the local kinematic outcomes of peak dorsiflexion and flexion angular velocity at the stance ankle and knee joints, respectively. The reduced peak dorsiflexion and flexion angular velocities in the U12 group could be a potential indication of increased leg stiffness during the stance phase of sprint running, which is associated with a maintained height of center of mass and good sprint running performance (Haugen et al., 2019). The increased force producing mass of the U12 group, as observed through increased vertical impulse, could have enabled the maintenance of a stiffer leg and increased energy return during the stance phase. Future research is required to examine the development of leg stiffness in boys by examining fine-grained local mechanics of the lower-limbs to help elucidate this point.

Sprinting and jumping ability are fundamental locomotive skills that form key aspects of athletic motor skill (Lloyd et al., 2015). The use of an effective countermovement is a distinguishing factor of skilled vertical jumping (Dowling and Vamos, 1993). The U12 group jumped significantly higher during the CMJ than the U9 group, which supported previous literature that investigated the CMJ jump performance across this age range (Focke et al., 2013). The added kinematic analysis of our study, which has previously not been concurrently investigated alongside a kinetic analysis, revealed that the U12 group had a reduced ROM at the ankle and a more constrained knee joint extension at take-off in the sagittal plane. The boys in the current study displayed greater ankle, knee and hip ROM when compared to a previous study of younger 3–10 years old boys and girls (Cowley et al., 2020), which may suggest an increase in unfreezing of the motor system degrees of freedom with skill development (Davids et al., 2000). However, the increased dorsiflexion at the bottom of the countermovement in the U9 group could be an indication of a lack of an appropriate proximal-distal sequencing of actions in their CMJ movement strategy. The successful performance of a CMJ requires the performer to assemble a technique that facilitates the development of high velocities in a proximal-distal temporal sequencing in the onset of joint movements. The increase in plantarflexion angular velocity and knee extension at take-off for the U9 group could be evidence of the participants attempt to correct for an exaggerated dorsiflexion of the ankle joint at the bottom of the countermovement phase. Therefore, unlike sprint running technique that may have already plateaued by the age of 8–9, CMJ technique may be still developing. To emphasize the lack of technique development in the CMJ, CMJ jump height has been suggested to not differentiate between elite youth soccer players and a control group at a similar age to the current study (10.9 ± 1.3 years), whereas 0–10 and 0–20 m sprint times have (Murtagh et al., 2018). Practitioners should be aware of the developing CMJ coordination patterns when using the CMJ as a physical assessment tool across the ages of 8–12 years old in boys.

During the CMJ, the suboptimal technique of the U9 group could limit the vertical velocity at take-off and, therefore, the height reached during the CMJ. The U12 group displayed greater average eccentric and concentric force, and increased time spent applying the propulsive force than the U9 group. The increased eccentric force during the countermovement would likely increase the activation of the stretch-shortening cycle (Harrison and Gaffney, 2001), enabling greater energy return in the U12 group. The eccentric action and the additional force generated during the concentric phase enabled an increased take-off velocity and jump height in the U12 group. Boys become gradually larger in skeletal length and width, as shown in our anthropometric results. Therefore, the main factors leading to better jumping performance in boys between the ages of 8–9 and 11–12 are potentially superior leg lengths, height, and CMJ technique. Practitioners could therefore utilize strength and conditioning interventions to increase muscle force producing capabilities to improve CMJ performance during this period of development.

A stepwise regression analysis was conducted in order to assess which key performance outcome, anthropometric, kinematic and kinetic variables best predicted the age of the participant during the performance plateau in boys between the ages of 8–12 years old. Our results suggested that average concentric force and 0–5 m sprint time predicted 93.7% of the variation. The regression supports the argument that the key developments during this period are the ability to generate and appropriately apply increased external force during dynamic movements. The findings of our study are unique and suggest that the cause for the plateau in sprint and CMJ performance are most likely a result of a stagnation in sprint running technique development and an underdeveloped CMJ technique that neither apply the increased force producing capabilities of the muscles appropriately to the ground. Future research requires the investigation into the longitudinal changes in sprint running and CMJ prior, throughout and beyond the plateau in sprint running and CMJ performance in order to further understand the mechanisms of the plateau in performance. One limitation of the current study is that although joint kinematic data were collected, only the first stance phase of an acceleration sprint run was observed. Previous literature by Nagahara et al. (2018) has examined the changes in GRF throughout the initial acceleration, middle acceleration and maximum velocity stages of sprint running but there is still a need for a kinematic analysis throughout each phase of the sprint run. A further limitation was that maturation was not controlled for within each group. It should be recognized that undertaking in depth kinematic and kinetic analysis in pediatric samples is more challenging than in adult samples and the value of the current study and future examinations lie in their comprehensive mechanical analysis undertaken and the unique value of this in informing effecting training, selection and talent identification programs.

## Conclusion

In summary, this is the first study to investigate the development of sprint running and CMJ kinematic and kinetics across the plateau in sprint running and CMJ performance in children. The study uniquely demonstrated that increased ground reaction forces and impulses were apparent in the older boys, but that sprint running technique had not developed during this period. Furthermore, CMJ technique was still emerging at the age of 8–9 years old. The findings highlight the need for on-going fine-grained sprint running and CMJ technique sessions to ensure that the increased force producing capabilities that come with age are appropriately utilized. Future research should expand on these findings by exploring the longitudinal changes in CMJ and sprint running technique.

## Data Availability Statement

The raw data supporting the conclusions of this article will be made available by the authors, without undue reservation.

## Ethics Statement

The studies involving human participants were reviewed and approved by the University’s Research Committee, CU Ethics and Governance, Coventry University. Written informed consent to participate in this study was provided by the participants’ legal guardian/next of kin. Written informed consent was obtained from the minor(s)’ legal guardian/next of kin for the publication of any potentially identifiable images or data included in this article.

## Author Contributions

MW and MD conceived the project. MW, MN, and MD collected the kinematic and kinetic data. MW and PM processed and analyzed the data. MW drafted the manuscript and other authors reviewed, edited, and gave final approval for publication. All authors contributed to the article and approved the submitted version.

## Conflict of Interest

The authors declare that the research was conducted in the absence of any commercial or financial relationships that could be construed as a potential conflict of interest.

## Abbreviations

CMJ: Countermovement jump
GRF: Ground reaction force
U9: 8–9 years old group
U12: 11–12 years old group
ROM: range of motion.
